# Supplementary material for: Automated Detection of Change of Direction in Basketball Players Using Xsens Motion Tracking
Source: Sensors (Basel). 2025 Feb 5;25(3):942. doi: 10.3390/s25030942 (PMC11820490; doi:10.3390/s25030942)
Supplement: Supplementary file 1 [file sensors-25-00942-s001.zip › sensors-3428245-supplementary.pdf]

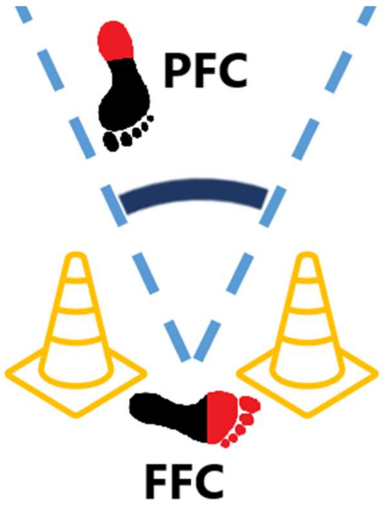

Figure S1 – Example of a 135° COD in a V-cut test. PFC: penultimate foot contact of non-cutting foot. FFC: final foot contact of cutting foot. In red, the contact points identify the PFC (heel) and FFC (toe).

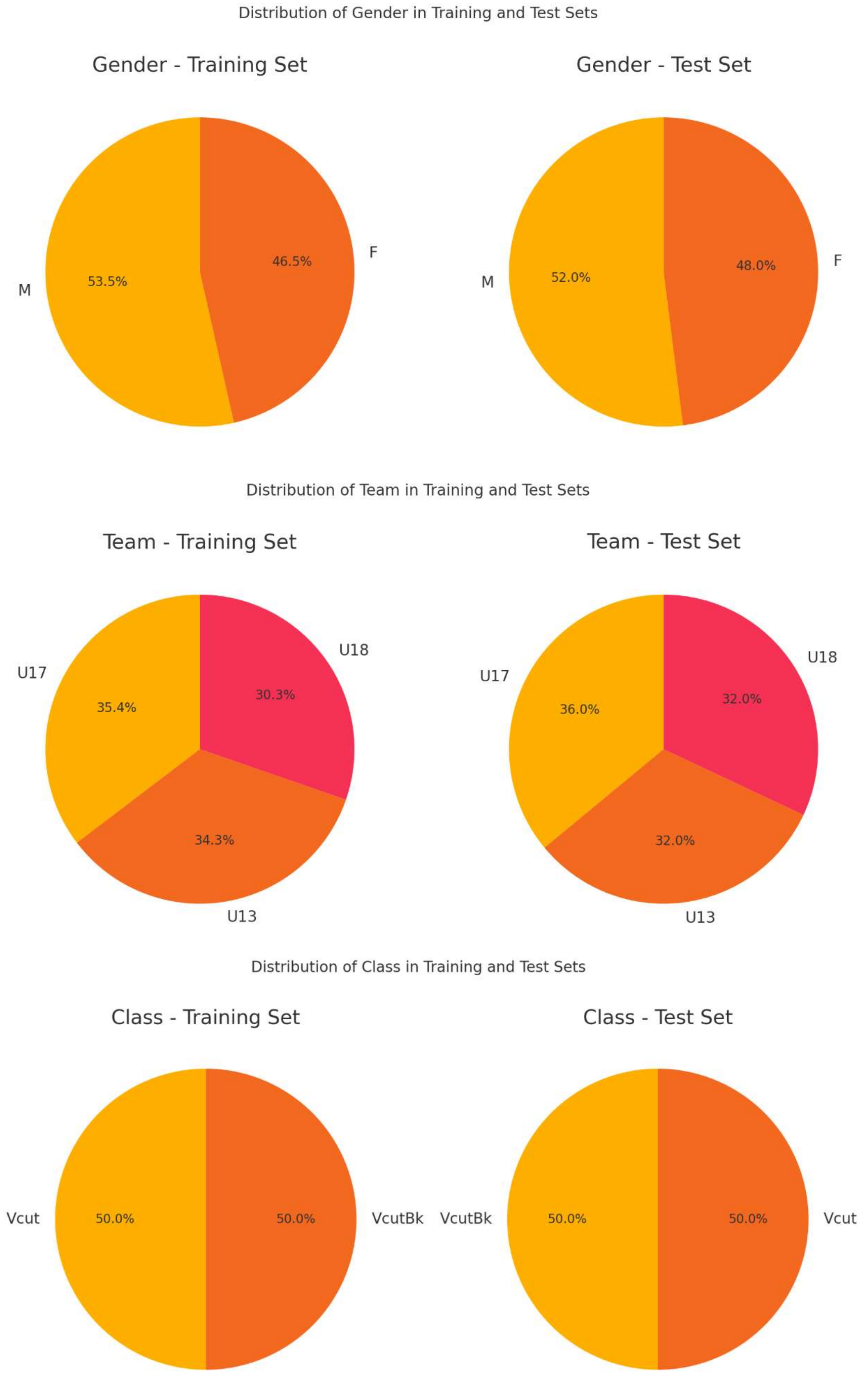

Figure S2 - Pie charts illustrating the percentage distribution of gender (M/F), team category (U13, U17, U18), and type of test (Vcut, VcutBk) in the training and test datasets.
